# Atypical Thymic Carcinoid Presenting as a Mediastinal Mass: A Case Report

**DOI:** 10.7759/cureus.93757

**Published:** 2025-10-03

**Authors:** Elgun Valiyev, İsmail Tombul, Tugce A Akbal Ersöz, Ulker Karagece, Muhammet Sayan

**Affiliations:** 1 Thoracic Surgery, Gazi University Hospital, Ankara, TUR; 2 Thoracic Surgery, Ankara Training and Research Hospital, Ankara, TUR; 3 Pathology, Ankara Training and Research Hospital, Ankara, TUR; 4 Thoracic Surgery, Gazi University, Ankara, TUR

**Keywords:** atypical carcinoid tumor, mediastinal mass, neuroendocrine tumors, thoracic surgery, thymectomy

## Abstract

Thymic neuroendocrine tumors are extremely rare and have generally been reported in the literature as individual case reports. Unlike their counterparts in other organs, thymic atypical carcinoid tumors usually exhibit aggressive behavior. Complete surgical resection is the recommended treatment. Herein, we present the case of a 70-year-old male patient who underwent surgery with a preliminary diagnosis of thymoma due to an anterior mediastinal mass and whose pathology was reported as a thymic atypical carcinoid tumor.

## Introduction

Neuroendocrine tumors are epithelial neoplasms that can arise in many organs, including the lungs, thymus, gastrointestinal tract, and ovaries [1]. In the 2021 World Health Organization classification of thymic tumors, thymic neuroendocrine tumors (tNETs) are very rare, accounting for about 5% of all thymic and mediastinal tumors with an estimated incidence of one per five million people, and are classified into four types: typical carcinoid, atypical carcinoid (AC), large cell neuroendocrine carcinoma (LCNEC), and small cell carcinoma (SCC). While their nomenclature traditionally follows that of pulmonary counterparts, low/intermediate-grade carcinoids (TC and AC) and high-grade carcinomas (LCNEC and SCC) are considered distinct entities rather than sequential stages of dedifferentiation [2,3]. Histological classification is based on the presence or absence of necrosis, mitotic activity, and large- or small-cell cytology. Prognosis is determined according to tumor stage, resectability, histological grade, proliferation index, and tumor size [4].

Except for lymphomas, primary mediastinal tumors are generally rare neoplasms. The diagnosis, classification, and treatment of these tumors, particularly primary thymic neuroendocrine carcinomas, pose challenges due to their low incidence and morphological heterogeneity [5]. They may arise from the thymus itself, from paraganglionic structures within the mediastinum, or as a result of neoplastic transformation in embryonic remnants in the mediastinum [6].

Herein, we present the case of a 70-year-old male patient who underwent surgery with a preoperative diagnosis of thymoma due to an anterior mediastinal mass and whose pathology was reported as a thymic atypical carcinoid tumor.

## Case presentation

**Figure 1:**
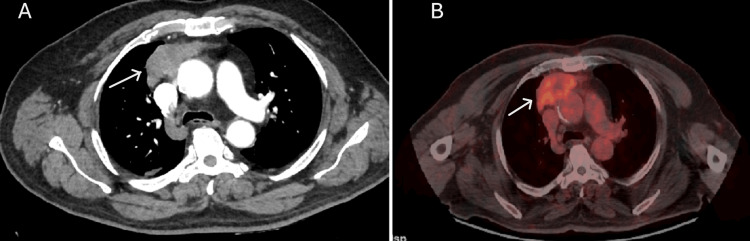
CT and PET/CT images of the anterior mediastinal mass CT (A) and PET/CT (B) images show a lobulated, irregularly marginated anterior mediastinal mass measuring 7.5 × 2.9 × 4.6 cm. The mass is inseparable from the pericardium and demonstrates focal obliteration of fat planes adjacent to the ascending aorta (white arrow). PET/CT shows increased F-18 fluorodeoxyglucose uptake (SUVmax: 5.91), indicating glucose metabolism in the tumor.

A 70-year-old male patient was admitted to the cardiology clinic with complaints of anterior chest pain. Initial evaluation to investigate cardiac causes included a 12-lead electrocardiogram and serum troponin measurement, both of which were within normal limits. A chest X-ray revealed mediastinal widening, and subsequent thoracic CT demonstrated a mass located in the anterior mediastinum, in the thymic lodge (65 × 34 × 49 mm) (Figure 1A).

PET/CT demonstrated a lobulated, irregularly marginated lesion in the anterior mediastinum, predominantly on the right side, measuring 7.5 × 2.9 × 4.6 cm at its largest dimension. The lesion was inseparable from the pericardium, with focal obliteration of fat planes adjacent to the ascending aorta, and exhibited increased F-18 fluorodeoxyglucose uptake (SUVmax: 5.91) (Figure 1B).

Due to its localization, the lesion was considered unsuitable for transthoracic biopsy, and since no additional uptake was detected on PET/CT outside the lesion, surgical excision was planned. Although definitive invasion of the lung parenchyma could not be appreciated on imaging, partial sternotomy was performed, and the mass was excised en bloc together with the invaded pericardial tissue and adjacent lung parenchyma. Surrounding mediastinal lymph nodes were also dissected.

**Figure 2:**
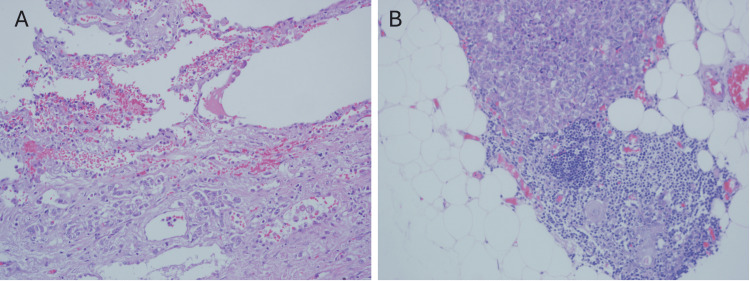
Histopathological invasion of the tumor Microscopic images demonstrate tumor invasion into the lung parenchyma (A, H&E, ×400) and thymic tissue (B, H&E, ×200). H&E staining highlights the atypical carcinoid morphology.

Histopathological examination revealed a thymic atypical carcinoid tumor. No lymph node metastasis was detected. Macroscopically, the tumor invaded the involuted thymic tissue, lung parenchyma, and surrounding adipose tissue (Figure 2A, 2B).

**Figure 3:**
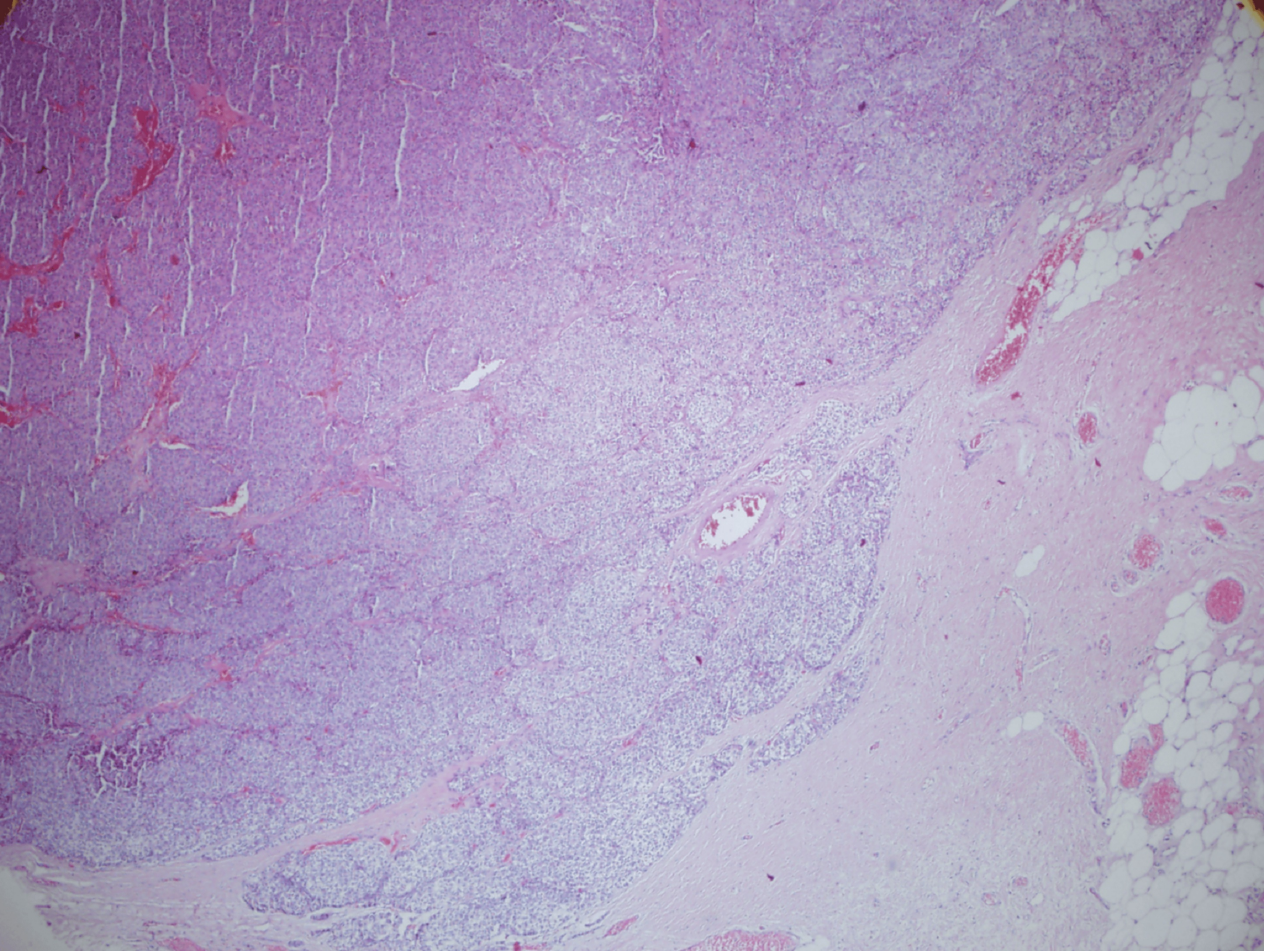
Microscopic morphology of the tumor Histopathological examination reveals nests of uniform neuroendocrine cells with granular chromatin and moderate cytoplasm (H&E, X40). Mitoses are rare, consistent with atypical carcinoid features.

Microscopically, the tumor was composed of atypical epithelial cells with large vesicular nuclei and salt-and-pepper chromatin, forming irregular solid nests, trabeculae, and occasional rosette-like structures (Figure 3).

Areas of necrosis and lymphatic invasion were also observed. The mitotic activity was 6-7/mm². The pericardial and pulmonary surgical margins were tumor-free, and no lymph node metastasis was present.

**Figure 4:**
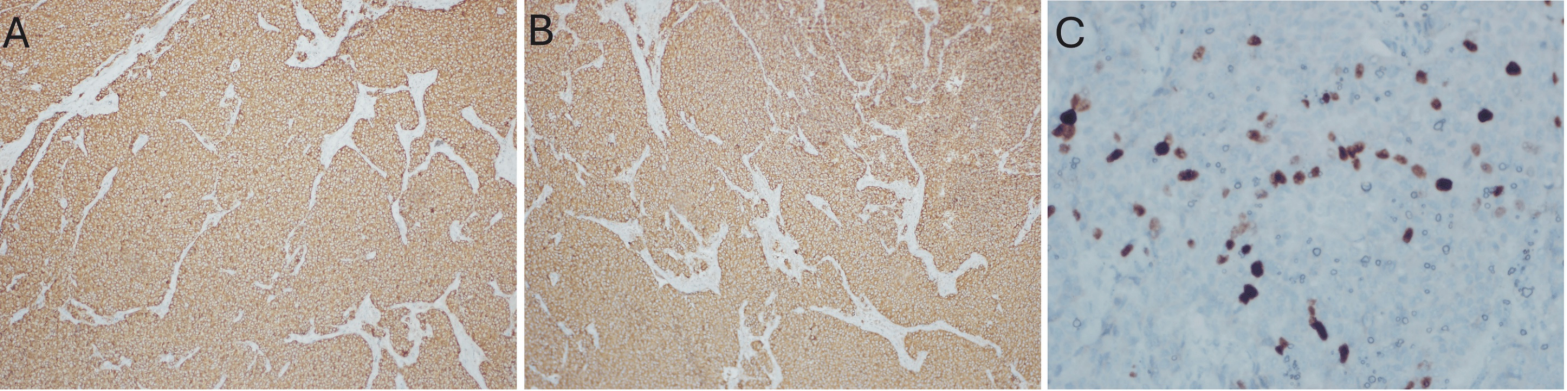
Immunohistochemical staining of the tumor Immunoreactivity of the tumor with different antibodies: (A, x100) diffuse chromogranin staining, (B, x100) positive CD56 staining, and (C, x400) Ki-67 proliferation index indicating tumor cell proliferation.

Immunohistochemical analysis showed strong positivity for chromogranin, CD56, and panCK, whereas TTF-1, CK7, CK20, S100, GATA3, calcitonin, CEA, and p53 were negative; the Ki-67 proliferation index was 15% (Figure 4A-4C).

Based on these findings, the patient was diagnosed with an atypical carcinoid tumor. The mediastinal carcinoid tumor measured 8.5 cm, invading the lung parenchyma and thymic tissue, with no lymph node involvement or distant metastasis. It was staged as T3N0M0 (Stage IIIA) according to the American Joint Committee on Cancer Eighth Edition.

Postoperative radiotherapy (50 Gray/25 fractions) was administered due to invasion. At the six-month postoperative follow-up, the patient remains recurrence-free under close surveillance with thoracic CT imaging.

## Discussion

The origin and classification of primary neuroendocrine tumors of the mediastinum remain a subject of debate due to the limited number of reported cases. The prognosis is poor, with a five-year survival rate of 0% and a median survival of 13.75 months (range: 13-26 months) [7-9]. tNETs are extremely rare, accounting for 2-5% of all thymic malignancies and approximately 0.4% of all neuroendocrine malignancies [4].

The etiology of tNETs is not yet fully understood. However, there is evidence suggesting a higher incidence of thymic carcinoid in patients with multiple endocrine neoplasia type 1 (MEN-1), particularly in heavy smokers [10]. In our case, MEN-1 was not present, although the patient had a 50 pack-year smoking history.

Among immunohistochemical markers, chromogranin is considered the most reliable neuroendocrine marker for these tumors, with chromogranin positivity reported in approximately 75% of cases. Additionally, co-expression of chromogranin and synaptophysin has been observed in about 60% of cases [11]. In our patient, diffuse chromogranin staining and focal weak synaptophysin positivity were identified.

Although rare, nearly one-third of thymic carcinoid tumors are associated with paraneoplastic syndromes [12]. In our patient, no paraneoplastic syndrome was observed.

Typical and atypical carcinoids of the thymus differ in terms of mitotic count and prognosis. Atypical carcinoid accounts for approximately 40-50% of tNETs, occurs predominantly in middle-aged adults (48-55 years), and up to 70% of patients are male [13]. Our patient, a 70-year-old male, was therefore consistent with the demographic characteristics reported in the literature.

For localized disease, complete surgical resection (R0) remains the preferred treatment. R0 resection is significantly associated with improved prognosis [14]. In our case, despite pericardial and pulmonary invasion, complete (R0) resection was achieved, with negative surgical margins and no distant metastasis.

To date, fewer than 300 cases of tNETs have been reported in the literature, of which approximately 30-40% are atypical carcinoids. Thus, the total number of anterior mediastinal atypical carcinoid cases is estimated to be around 100-120 [15].

## Conclusions

Atypical carcinoid tumors located in the anterior mediastinum are exceedingly rare epithelial neoplasms. This case highlights the importance of surgical resection and the decisive role of histopathological confirmation in establishing the diagnosis. The presence of such tumors should always be considered in the evaluation of mediastinal masses. Although clinical and molecular data on tNETs are limited, their behavior and genetic makeup appear similar to corresponding neuroendocrine tumors in other organs. Consequently, the classification of tNETs is likely to continue aligning with emerging concepts in other organ systems. However, many questions regarding patient management and therapy remain unresolved, and further studies are required to elucidate the immunohistochemical, molecular, and genetic peculiarities of these tumors.
